# Clinical, Hematological, and Nutritional Profiling of Thalassemia Patients: Insights From a Cross‐Sectional Study

**DOI:** 10.1155/anem/8514650

**Published:** 2026-09-23

**Authors:** Md. Rakibul Hasan Rahat, Md. Imtiajul Haque, Sinthia Doly Shurmi, Akbor Hossain, Muzahid Fahim, Arafat Bin Muhammad, Nur Islam Bahar, Md. Earul Islam, Razibul Islam, Md Kamruzzaman, Shaikh Shahinur Rahman

**Affiliations:** ^1^ Department of Applied Nutrition and Food Technology, Faculty of Biological Sciences, Islamic University, Kushtia 7003, Bangladesh, iu.ac.bd; ^2^ Upazila Health Complex, Sarsha, Jashore, Bangladesh; ^3^ Doctor’s Lab, Diagnostic and Hospital, Kushtia 7000, Bangladesh; ^4^ Unani Medicine, Bikolpo Treatment Center, Jhenaidah 7300, Bangladesh

**Keywords:** Bangladesh, cross-sectional study, dietary habits, hemoglobin, thalassemia

## Abstract

**Background:**

Thalassemia is a significant inherited hemoglobin (Hb) disorder that poses a major public health burden in Bangladesh. While the role of nutrition and lifestyle in disease management is now recognized, there is still little data on the sociodemographic, clinical, nutritional, and lifestyle factors of thalassemia patients and their relationship with Hb levels. The aim of this study was to describe these factors and identify independent predictors of Hb levels in individuals with thalassemia in Bangladesh.

**Methods:**

This was a multicenter cross‐sectional study of 270 previously diagnosed thalassemia patients from thalassemia care facilities in Bangladesh. Hb electrophoresis was used for the diagnosis of all participants, but no genotype subtype was recorded. Sociodemographic, clinical, dietary, and lifestyle information was gathered, and blood samples were tested for Hb levels. Descriptive statistics were used to summarize participant characteristics, and multivariable linear regression was used to determine the independent predictors of Hb levels after adjusting for potential confounders.

**Results:**

The participants had a mean age of 13.9 ± 10.8 years; 55.0% were male, and 93.0% were Muslim. The mean BMI was significantly low (15.8 ± 3.4 kg/m^2^), and the mean Hb concentration was severely low (7.5 ± 1.3 g/dL). Dietary quality was poor, with high consumption of junk food (98.9%) and trans fat (99.6%), high consumption frequency of raw salt (94.1%), and low consumption frequency of vegetables (5.9%). The multivariate model was not statistically significant (*R* ^2^ = 0.111; *F* (21, 248) = 1.47; *p* = 0.089). Daily fruit intake was inversely associated with Hb levels (*β* = −0.76; 95% CI: −1.30 to −0.21; *p* = 0.007); however, this association was not significant in the unadjusted analysis and was not significant after multiple testing. The apparent association with trans‐fat intake was not interpretable since only one participant reported avoiding trans‐fat. Hb levels were not significantly related to any other demographic, clinical, or dietary variable.

**Conclusion:**

Thalassemia patients in Bangladesh were found to be severely anemic, unduly undernourished, and with poor dietary quality. The assessed factors accounted for little variation in Hb levels, and none of the factors were associated after multiple‐testing correction. These findings emphasize the importance of adequate transfusion management and nutrition in these participants.

## 1. Introduction

Thalassemia is one of the most common inherited hemoglobin (Hb) disorders worldwide, characterized by mutations in the alpha‐ or beta‐globin genes that result in deficient or absent globin chain synthesis, leading to chronic hemolytic anemia, ineffective erythropoiesis, and a cascade of systemic complications [1]. Recognized by the World Health Organization (WHO) as a major global health concern, thalassemia has historically been most prevalent in the Mediterranean, Middle East, and Southeast Asia, although population migration has progressively altered its global distribution. According to a 2008 WHO report, over 40,000 babies are born with beta‐thalassemia annually, with approximately 25,500 of these cases being transfusion‐dependent, and outcomes differ markedly between high‐ and low‐income countries [2]. The prevalence of thalassemia genetic mutations among carriers is estimated to be between 1.5% and 7% of the world’s population, with the high incidence of inherited Hb variants in certain regions attributed in part to heterozygote resistance to Plasmodium falciparum malaria [3].

Bangladesh bears a disproportionately higher burden of thalassemia, with a reported carrier prevalence of 10.9%–13.3%, translating to approximately 17–22 million carriers, with significant regional and ethnic variations. Most cases of transfusion‐dependent thalassemia in Bangladesh involve Hb E‐Beta thalassemia [4]. National screening data from school children across all six divisions of Bangladesh found an overall prevalence of beta‐thalassemia trait of 4.1% and Hb E trait of 6.1%, with thalassemia anticipated to become a major genetic public health problem in the coming years [4, 5]. In Bangladesh, approximately 33 per 10,000 newborns are estimated to be born with thalassemia each year; however, thalassemia patient care and support facilities are barely available in most public and private hospitals, and awareness of thalassemia remains highly inadequate among the general population [6].

Transfusions continue to be an important component of the ongoing management of persons with moderate or severe forms of thalassemia. It is a therapy that increases life expectancy significantly, but if not managed well, it can lead to several secondary complications, the most important of which is iron overload, which progressively affects the liver, heart, and endocrine organs [7]. During the past 50 years, progress in hemovigilance, iron chelation, and multidisciplinary care has changed the prognosis for patients with transfusion‐dependent β‐thalassemia. However, access to these advances and the optimal use of best practices is still largely limited to high‐income countries. Multimorbidity and shortened survival continue to be a burden to patients in low‐resource countries where most affected patients live [8]. Poor adherence to iron chelation therapy has been noted among adolescents with transfusion‐dependent thalassemia in low‐income families, with significant implications for clinical management in populations that cannot afford chelating agents [9]. Beyond core hematological management, the nutritional dimension of thalassemia care has received limited attention, particularly in low‐ and middle‐income countries. Patients with thalassemia exhibit circulating deficiencies in most routinely assessed vitamins and minerals, with deficiency prevalence exceeding 40% for vitamin A, vitamin D, selenium, and zinc; however, there are no universally accepted nutrition consensus statements to guide the standard of care. The etiology of nutritional inadequacy in thalassemia is multifactorial, relating to a combination of poor nutrient intake driven by poor appetite, cultural preferences, and decreased nutrient density, alongside increased nutrient requirements due to red blood cell turnover and oxidative stress, and increased nutrient loss or sequestration [10].

In spite of the increasing concern about thalassemia, data on adults (especially beyond adolescence) are still relatively scarce. Most of the data in Bangladesh show a high prevalence of thalassemia carriers, but regional data are lacking. Despite the common acknowledgment of nutritional vulnerability in thalassemia, there is no evidence from Bangladesh that specifically addresses dietary habits, lifestyle behaviors, and association with Hb levels in this population. It is important to understand these factors because Hb level is a key surrogate for disease control, adequacy of transfusion, and general clinical well‐being. Moreover, identifying modifiable dietary and lifestyle predictors of Hb levels offers the prospect of practical, low‐cost adjunctive interventions that could meaningfully improve patient outcomes in a resource‐constrained healthcare system. The present study aimed to characterize the demographic and clinical profiles, dietary habits, and lifestyle factors of individuals with thalassemia in Bangladesh and to identify the independent predictors of Hb levels through multivariable regression analysis.

## 2. Materials and Methods

### 2.1. Study Design

In this cross‐sectional observational study, individuals with previously diagnosed thalassemia were consecutively recruited from one medical college hospital and one general practitioner clinic in both the urban and rural parts of the Khulna Division, Bangladesh, where thalassemia management is available. Uniformity of clinical evaluation and laboratory assessment was achieved by performing data collection over a 1‐year study period. A convenience sampling procedure was used to select participants with thalassemia; no a priori sample size calculation or power analysis was undertaken, and the achieved sample of 270 comprised all eligible patients who presented during the recruitment period from April 2022 to June 2023. Individuals with previously diagnosed thalassemia confirmed via blood analysis and hematologic and Hb variant studies were included. Participants with acute infections, known chronic inflammatory or malignant illnesses, history of blood transfusions within 3 months prior to recruitment, and any other event that may have caused changes in hematologic parameters were excluded. The purpose of the study and details of the study procedures were first explained to the participants and their guardians. Written informed consent was obtained from all adult participants (≥ 18 years). For participants younger than 18 years, written consent was obtained from a parent or legal guardian, and assent was obtained from children aged 7–17 years old. Standardized tools were used to perform all measurements in the study, and the tools were pretested before the field visit. The questionnaires were reviewed and corrected for completeness and internal consistency prior to data entry. The study protocol was approved by the Ethics Committee of the Faculty of Biological Sciences of the Islamic University, Kushtia, Bangladesh (Ref. No. ERC/FBS/IU/2022/02).

### 2.2. Data Collection

During the direct interview, a structured questionnaire was used to collect comprehensive demographic and clinical information from participants. Anthropometric measurements, including weight and height, were performed on the day of recruitment using a clinically calibrated weight scale and a stadiometer. BMI was calculated using the standard equations of the WHO reference criteria [11].

### 2.3. Hematological and Clinical Information

Blood samples (3–5 mL) were collected on the day of recruitment by a trained professional under aseptic conditions. For hematological analysis, samples were collected in an EDTA tube to minimize analytical variability, and all samples were processed on the day of collection. An automated hematology analyzer was used following the standard operating procedures (SOPs) provided by the manufacturer to analyze routine hematological parameters. Hematological parameters, including blood Hb levels, were analyzed. Blood group typing and agglutination techniques were used to confirm thalassemia diagnosis. Information related to the reports of patients and caregivers about blood transfusion, volume and time of transfusion, current medications, and folic acid supplements was systematically documented.

### 2.4. Assessment of Dietary Habits and Lifestyle Factors

A standardized, structured questionnaire was used to assess the dietary practices and lifestyle characteristics of the participants. Intake items were recorded as categorical or binary responses rather than quantified as portion sizes. Information on daily meal frequency, fruit and vegetable intake, junk food consumption, and intake of saturated fat, trans fat, and raw salt was collected. The daily routine activity levels of physical activity were quantitatively assessed.

### 2.5. Statistical Analysis

Statistical analyses were conducted using RStudio statistical software based on R (v4.6). Continuous variables are presented as means ± standard deviations. Categorical variables are presented as counts and percentages. Multivariable linear regression was used to examine the association between Hb level (g/dL) and sociodemographic, clinical, dietary, and lifestyle characteristics. Candidate predictors were selected a priori based on clinical and biological plausibility and prior literature rather than by data‐driven stepwise procedures, and all prespecified covariates were retained in the final model. Model assumptions were examined using standard residual diagnostics: linearity and homoscedasticity from residual‐versus‐fitted plots and the Breusch–Pagan test (LM = 13.28; *p* = 0.898; satisfied), normality of residuals from a normal quantile–quantile plot and the Shapiro–Wilk test (*W* = 0.948; *p* = 3.1 × 10^−8^), and multicollinearity from variance inflation factors (all < 1.5; satisfied) (Supporting Table S1). The residuals departed from normality, and the principal dietary coefficient was therefore additionally examined using heteroscedasticity‐consistent (HC3) standard errors and a 5000‐replicate bootstrap analyses. Overall model performance is reported as *R* ^2^, adjusted *R* ^2^, and the *F*‐statistic with its *p* value. Variables that were invariant in this cohort (caffeinated beverage intake, folic acid supplementation, and low physical activity, each 100%) provided no contrast, were not estimable, and were therefore excluded from the model and reported descriptively. Four dietary predictors were near invariant, with reference groups of one (trans fat), two (added sugar), and three (junk food, saturated fat) participants. These were retained in the prespecified model for transparency, but their coefficients were uninterpretable as effect estimates. Therefore, two prespecified sensitivity analyses were performed: one excluding all four near‐invariant predictors and one excluding the single participant who reported avoiding trans fat. Because 21 terms were estimated, *p* values were additionally examined under the Benjamini–Hochberg false discovery rate control (Supporting Table S2). No missing data were identified. All 270 participants had complete data for all variables. Statistical significance was set at *p* ≤ 0.05.

## 3. Results

**FIGURE 1:**
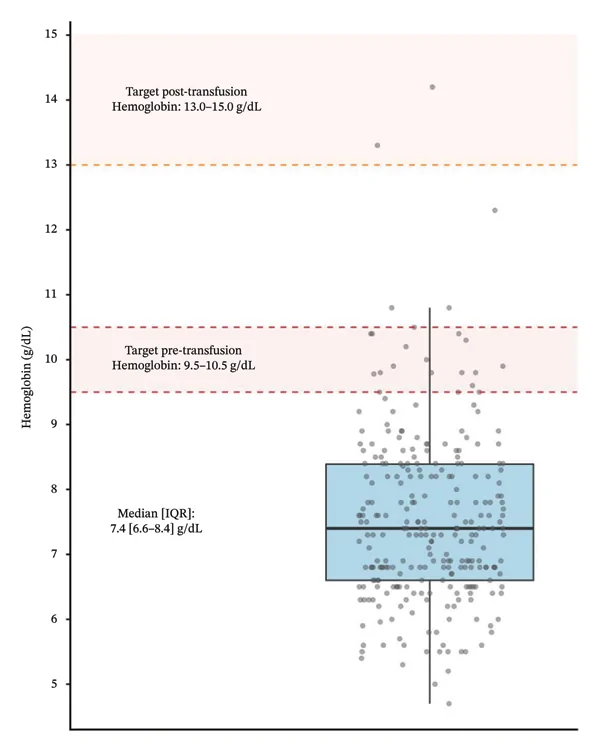
Hemoglobin distribution according to international transfusion targets (*n* = 270). Box denotes median (7.4 g/dL) and interquartile range (6.6–8.4 g/dL), whiskers 1.5 × IQR, with individual values shown as jittered points. Dashed lines mark the Thalassemia International Federation (TIF) pre‐transfusion (9.5–10.5 g/dL, red) and post‐transfusion (13.0–15.0 g/dL, orange) targets [8]. The mean hemoglobin level was 7.5 ± 1.3 g/dL; over three‐quarters of the participants fell below the pre‐transfusion target.

The demographic and clinical characteristics of the 270 individuals with thalassemia are shown in Table 1. The participants of this study were predominantly younger, with a mean age of 13.9 ± 10.8 years, suggesting that a large portion of the sample consisted of children and adolescents. Slightly less than half of the participants were female (45.0%), and the vast majority identified as Muslim (93.0%). In terms of anthropometric measures, the mean weight was 28.0 ± 14.5 kg and mean height was 4.2 ± 1.1feet, with a notably low mean BMI of 15.8 ± 3.4 kg/m^2^. Blood Hb levels were also markedly reduced across the cohort (median 7.4 g/dL; IQR 6.6‐8.4; range 4.7–14.2 g/dL). Only 18 participants (6.7%) fell within the recommended pretransfusion target of 9.5–10.5 g/dL, whereas 247 (91.5%) were below and five (1.9%) were above this range. Only two participants (0.7%) reached the recommended posttransfusion Hb range of 13.0–15.0 g/dL (Figure 1). Regarding blood group distribution, B+ was the most prevalent (35.6%), followed by O+ (30.4%) and A+ (24.1%), whereas the remaining groups were relatively rare. Clinically, hepatomegaly was present in nearly all participants (93.7%), and splenomegaly was also common (88.0%). Jaundice was reported in 12.0% of cases. All 270 participants (100%) received folate supplementation, and approximately one in eight (12.0%) had a family history of Thalassemia. The majority of participants (88.9%) received blood transfusions, while 10.7% required them twice, and only 0.37% required transfusions three times.

**TABLE 1:** Characteristics of individuals with thalassemia in Bangladesh (*n* = 270).

	Mean ± SD or *n* (%)
Age, years	13.9 ± 10.8
Female	122 (45.0)
Muslim	252 (93.0)
Weight, kg	28.0 ± 14.5
Height, feet	4.2 ± 1.1
BMI, kg/m^2^	15.8 ± 3.4
Hemoglobin, g/dL	7.5 ± 1.3
Blood group	
A+ve	65 (24.1)
AB−ve	2 (0.7)
AB + ve	21 (7.8)
B−ve	2 (0.7)
B+ve	96 (35.6)
O−ve	2 (0.7)
O+ve	82 (30.4)
Jaundice	33 (12.0)
Hepatomegaly	253 (93.7)
Splenomegaly	238 (88.0)
Folic acid supplementation	270 (100.0)
Family history of thalassemia	32 (12.0)
Blood intake by transfusion	
Once	240 (88.89)
Twice	29 (10.74)
Thrice	1.00 (0.37)

The dietary habits and lifestyle characteristics of the participants are presented in Table 2. The majority of participants ate three meals per day (97.8%), with only a small fraction consuming four (1.9%) or as few as two meals per day (0.37%). Raw salt consumption was reported by 94.1% of the participants, and junk food intake was nearly universal (98.9%). Daily fruit intake was reported by 90.0% of participants, whereas nearly all participants reported trans‐fat intake (99.6%). Vegetable intake was recorded at two levels and was predominantly low: 253 participants (93.7%) reported low intake and 17 (6.3%) reported moderate intake. All 270 patients (100%) reported low physical activity. Pallor, reduced appetite, caffeinated beverage consumption, and folic acid supplementation were universal (100%).

**TABLE 2:** Dietary habits, lifestyle factors, and nutritional awareness.

	*n* (%)
Eating per day	
2 times	1 (0.37)
3 times	264 (97.78)
4 times	5 (1.85)
Raw salt intake	254 (94.1)
Junk food intake	267 (98.9)
Saturated fat intake	267 (98.9)
Daily fruit intake	242 (90.0)
Take trans fat	269 (99.6)
Added sugar intake	268 (99.3)
Daily fruit intake	242 (89.6)
Caffeinated beverage intake	270 (100.0)
Vegetables intake	
Low	253 (93.70)
Moderate	17 (6.3)
Medium	1 (0.37)
Regular	16 (5.93)
Lower physical activity	270 (100.0)

The overall model explained 11.1% of the variance in Hb (*R* ^2^ = 0.111; adjusted *R* ^2^ = 0.035) and was not statistically significant (*F* (21, 248) = 1.47; *p* = 0.089). The multivariate linear regression outcomes for predicting Hb levels among participants with thalassemia in relation to demographic, clinical, and dietary factors are shown in Table 3. Age was not significantly negatively associated with Hb levels (*β* = −0.01; 95% CI: −0.02, 0.01; *p* = 0.457). Similarly, sex did not emerge as a significant predictor, with females showing a slightly higher *β* coefficient than males (*β* = 0.17; 95% CI: −0.16, 0.50; *p* = 0.307). Religion and BMI were also not significantly associated with Hb levels (*p* = 0.643 and *p* = 0.267, respectively). None of the blood group categories demonstrated a statistically significant association with Hb levels when compared with the B+ reference group (*p* values ranging from 0.159 to 0.603). Jaundice did not reach conventional statistical significance (*β* = −0.48; 95% CI: −0.98, 0.03; *p* = 0.065). Hepatomegaly, splenomegaly, daily eating frequency, raw salt intake, junk food intake, saturated fat intake, and added sugar intake were not statistically significant in the multivariable model. However, two dietary variables were independently and significantly associated with Hb levels. Trans fat intake was negatively and significantly associated with Hb levels (*β* = −3.0; 95% CI: −5.6, −0.39; *p* = 0.024), indicating that patients who consumed trans‐fats had considerably lower Hb levels. This corresponds to a mean Hb level 3.0 g/dL lower among trans‐fat consumers, but the estimate is highly imprecise: only one participant reported avoiding trans‐fat, so the comparison rests on a single observation and the coefficient should not be interpreted as a reliable effect size. Daily fruit intake was also significantly and negatively associated with Hb levels (*β* = −0.76; 95% CI: −1.3, −0.21; *p* = 0.007). This equates to a mean Hb level 0.76 g/dL lower among daily fruit consumers than among nonconsumers, as only 28 participants (10.0%) reported nondaily intake. Low vegetable intake showed a negative beta coefficient compared with the moderate reference category, although this did not reach statistical significance (*β* = −0.56; 95% CI: −1.2, 0.12; *p* = 0.106). Two features of these estimates require emphasis. First, the trans‐fat coefficient rests on a single participant, the only individual reporting no trans‐fat intake; this observation has a leverage of 1.000 and an undefined Cook’s distance, meaning the model reproduces it exactly and the coefficient is an interpolation through one data point rather than an estimated association. Second, in sensitivity analyses, the daily‐fruit coefficient was essentially unchanged when the four near‐invariant dietary variables were removed (*β* = −0.76; 95% CI: −1.31, −0.22; *p* = 0.006) and when the single trans‐fat nonconsumer was excluded (*β* = −0.77; 95% CI: −1.31, −0.23; *p* = 0.006) (Supporting Table S2). However, the fruit‐Hb difference was not significant in unadjusted comparisons (Welch *t*‐test *p* = 0.064; Mann–Whitney *p* = 0.100), attenuated under HC3 robust standard errors (*p* = 0.037), and did not survive false discovery rate correction (adjusted *p* = 0.145), as was the case for every other coefficient in the model.

**TABLE 3:** Multivariable linear regression analysis of factors associated with hemoglobin levels among individuals with thalassemia in Bangladesh.

Characteristic	Beta	95% CI	*p* value
Age	−0.01	−0.02, 0.01	0.457

*Sex*			
Male	Ref.	—	
Female	0.17	−0.16, 0.50	0.307

*Religion*			
Islam	Ref.	—	
Hindu	0.15	−0.48, 0.78	0.643
BMI	0.03	−0.02, 0.08	0.267

*Blood group*			
B+ve	Ref.	—	
A+ve	−0.15	−0.60, 0.29	0.497
AB−ve	−0.71	−2.6, 1.2	0.455
AB+ve	−0.46	−1.1, 0.18	0.159
B−ve	−0.49	−2.4, 1.4	0.603
O−ve	−1.1	−3.0, 0.79	0.252
O+ve	0.18	−0.22, 0.58	0.376

*Jaundice*			
No	Ref.	—	
Yes	−0.48	−0.98, 0.03	0.065

*Hepatomegaly*			
No	Ref.	—	
Yes	0.26	−0.43, 0.95	0.460

*Splenomegaly*			
No	Ref.	—	
Yes	0.03	−0.47, 0.54	0.893
Daily eating frequency	0.26	−0.82, 1.3	0.636

*Raw salt intake*			
No	Ref.	—	
Yes	0.41	−0.30, 1.1	0.252

*Junk food intake* ^†^			
No	Ref.	—	
Yes	0.42	−1.1, 2.0	0.592

*Saturated fat intake* ^†^			
No	Ref.	—	
Yes	−0.84	−2.4, 0.73	0.293

*Trans fat intake* ^†^			
No	Ref.	—	
Yes	−3.0	−5.6, −0.39	**0.024**

*Added sugar intake* ^†^			
No	Ref.	—	
Yes	−0.10	−2.0, 1.8	0.920

*Eat fruits daily*			
No	Ref.	—	
Yes	−0.76	−1.3, −0.21	**0.007**

*Vegetable intake*			
Moderate	Ref.	—	
Low	−0.56	−1.2, 0.12	0.106

*Note:* Overall model: *R* ^2^ = 0.1106; adjusted *R* ^2^ = 0.0353; *F* (21, 248) = 1.47; *p* = 0.089. The overall model was not statistically significant and explained 3.5% of the variance after adjustment. The trans‐fat estimate had leverage = 1.000 and an undefined Cook’s distance; that is, it was fitted through a single observation and was not an interpretable effect estimate. Under the Benjamini–Hochberg correction across the 21 terms, no coefficient retains significance (fruit FDR *p* = 0.145; trans‐fat FDR *p* = 0.257). Bold values indicate statistically significant with *p* value < 0.05.

Abbreviation: CI, confidence interval.

^†^Near‐invariant predictor: the reference group contained 1 (trans fat), 3 (junk food), 3 (saturated fat), and 2 (added sugar) participants.

## 4. Discussion

The current study provides a detailed insight into the demographic, clinical, and dietary characteristics, lifestyle behaviors, and factors related to Hb levels in individuals with thalassemia in Bangladesh. In this study, the Hb levels of individuals with thalassemia were found to be significantly lower than those of individuals with thalassemia in other countries. The demographic, clinical, and dietary variables measured showed no correlation with Hb levels, and the multivariable model was not significant overall. Daily fruit intake was the only variable associated with Hb levels; however, this association did not persist after adjusting for multiple variables.

The average Hb level was 7.5 g/dL found in the current study represents a clinically critical level of anemia, particularly in young children and adolescents. Current guidelines for the management of thalassemia recommend maintenance of pretransfusion Hb levels above 9.5 g/dL to support normal growth and development, with persons affected by beta‐thalassemia major requiring periodic and lifelong blood transfusions to sustain Hb above this threshold [7, 12]. Beyond survival, chronic Hb levels below the threshold may profoundly affect oxygen delivery to peripheral tissues and developing organs. Untreated or inadequately managed childhood anemia has the potential to cause chronic and irreversible cognitive impairment, resulting in poor academic performance and potential reduction in work capacity in adulthood [13]. In the context of thalassemia, these effects are further compounded by ineffective erythropoiesis. Chronic, severe anemia in individuals with thalassemia may result in bone marrow expansion and extramedullary hematopoiesis, contributing to the skeletal deformities, hepatosplenomegaly, and growth retardation widely observed in inadequately transfused patients [14]. Taken together, the lower Hb levels recorded in this cohort reflect an acute unmet therapeutic need and signal the risk of cumulative, potentially irreversible morbidity in a population already burdened by the systemic complications of thalassemia.

The cohort was predominantly young, with a mean age of 13.9 ± 10.8 years, reflecting the well‐established pattern that thalassemia typically manifests and is diagnosed in childhood and early adolescence. This is consistent with national‐level data from Bangladesh, where thalassemia, particularly Hb E‐Beta thalassemia, disproportionately affects the pediatric population and accounts for most transfusion‐dependent cases. In Bangladesh, more than 70% of patients with thalassemia are diagnosed with Hb E‐Beta thalassemia, with beta thalassemia major constituting less than 30% of cases [5]. The slight male predominance observed in this study aligns with the findings of hospital‐based surveys in Bangladesh, where male patients consistently outnumber females among anemic children with hemoglobinopathies, with a male‐to‐female ratio of approximately 1.45:1 [15]. The overwhelming majority of patients identified as Muslim (93.0%), which reflects the broader demographic composition of Bangladesh, where consanguineous marriages, a known risk factor for autosomal recessive conditions such as thalassemia, remain culturally prevalent in certain communities [16].

The markedly low mean BMI of 15.8 ± 3.4 kg/m^2^ in the current cohort points to widespread undernutrition in this population. This finding corroborates previous studies. Undernutrition, defined as a low weight‐for‐age z‐score, low BMI z‐score, or low weight‐for‐length z‐score, occurs in a considerable proportion of children and adolescents with beta‐thalassemia major, with prevalence estimates ranging from 20% to 71% across different countries [17]. ^2^Another study conducted in India showed that the mean BMI of patients with thalassemia major was 13.9 ± 1.6 kg/m2, and 48.25% of the patients were underweight [18].

The high levels of hepatomegaly (93.7%) and splenomegaly (88.0%) are consistent with the disease pathology, as organ hypertrophy due to iron deposition and ineffective erythropoiesis are well documented [19]. Hepatomegaly is prominent early in thalassemia due to increased red cell destruction and extramedullary erythropoiesis in the liver and tends to be more pronounced in children [7, 20]. Similarly, splenomegaly occurs because the spleen removes faulty red blood cells, and both the liver and spleen serve as alternative sites for red blood cell production, with a markedly enlarged spleen capable of worsening the anemia. Hepatomegaly and splenomegaly have also been linked to lower pretransfusion Hb levels, particularly in patients with Hb E/β‐thalassemia [21]. The high rate of blood transfusion dependency (88.9% requiring transfusions at least once) and universal folate supplementation observed in this study are consistent with the standard management protocols for transfusion‐dependent thalassemia in resource‐limited settings [22].

Although the majority of participants claimed to eat three meals a day, details on the quality of the meals are required to fully elucidate the potential effects. The near‐universal consumption of junk food (98.9%) and trans fat (99.6%), alongside raw salt intake in 94.1% of patients, represents a dietary quality profile that is deeply incompatible with optimal hematological health. Frequent consumption of junk food, which is characteristically deficient in essential micronutrients, including iron, folate, and vitamin C, has been associated with impaired Hb synthesis and compromised iron absorption [23, 24]. The exceedingly low proportion of patients with regular vegetable intake (5.93%) is particularly alarming, as vegetables are important sources of folate and other micronutrients essential for erythropoiesis. Nutritional deficiencies in thalassemia are common due to hemolytic anemia, increased nutritional requirements, and comorbidities such as iron overload, diabetes, and chelation therapy use, with annual evaluation by a registered dietitian recommended for all patients [10]. Notably, poor dietary practices alongside socioeconomic constraints, limited food availability, and the demands of managing a chronic illness plausibly shape dietary behavior in this population.

In the multivariable regression analysis (Table 3), two dietary variables reached nominal significance, but neither could bear the interpretive weight that was originally placed on them. The overall model was not significant (*F* (21, 248) = 1.47; *p* = 0.089) and explained 3.5% of the variance in Hb after adjustment, and under false discovery rate control, no coefficient retained significance. The near‐zero variance in trans‐fat intake (269 of 270 participants reported consuming trans‐fat) was the cause of the apparent association with Hb (*β* = −3.0; *p* = 0.024). The coefficient was estimated from a single nonconsumer, whose Hb of 10.4 g/dL was among the highest recorded in the cohort. This observation has a leverage of 1.000, meaning that the model reproduces it exactly as it is. Therefore, we no longer regarded trans‐fat intake as a finding in this study. Trans‐fat intake remains a recognized population health risk, and the WHO recommends limiting it to less than 1% of total energy intake [25]. Beyond cardiovascular risk, trans fat‐rich diets are characteristically proinflammatory [26], and chronic low‐grade inflammation has been recognized as a suppressor of erythropoiesis through mechanisms, including hepcidin upregulation [27], which restricts iron availability for Hb synthesis. However, no inflammatory, iron status, or dietary fat biomarkers were measured in this study; therefore, this pathway was not tested and is offered only as a candidate explanation. Given that only one participant reported avoiding trans fat, the magnitude of this coefficient should not be over‐interpreted. The negative association between daily fruit intake and Hb levels (*β* = −0.76; *p* = 0.007) is a nuanced finding that warrants careful interpretation. While fruits are broadly considered nutritious, for patient with thalassemia already at risk of iron overload, the high vitamin C content of many fruits can paradoxically enhance intestinal iron absorption. Polyphenols found in fruits and vegetables have been shown to inhibit iron absorption; however, ascorbic acid, which is abundant in citrus and tropical fruits, promotes nonheme iron absorption by converting ferric iron to ferrous iron [28]. In the context of thalassemia, where dysregulated iron metabolism is central to disease pathophysiology, vitamin C intake can significantly impact iron absorption, and thalassemia patients should carefully balance their vitamin C intake to avoid unnecessary worsening of iron overload [10]. The inverse association observed here may therefore reflect the deleterious effects of unmonitored high–vitamin C fruit consumption in the setting of an already elevated iron burden, although this interpretation should be regarded as hypothesis‐generating rather than established. Notably, fruit intake was captured only as a binary variable, without information on type, quantity, or vitamin C content, and approximately 90% of the participants reported daily consumption, leaving a small comparison group that may yield unstable estimates. Therefore, the observed inverse association requires confirmation in larger studies using quantitative dietary recall and iron‐status biomarkers before any mechanistic or clinical inferences are drawn. Because vitamin C intake, iron status, and iron overload were not measured, the ascorbate–iron pathway outlined above was not evaluated in this study and is offered solely as a direction for future research rather than as an explanation of the present finding. No statistically significant associations were observed for age, sex, religion, BMI, blood group, hepatomegaly, splenomegaly, eating frequency, raw salt, junk food, saturated fat, or added sugar intake in this multivariable model, suggesting that the dietary fat composition, particularly trans‐fat rather than overall dietary pattern or clinical characteristics, was the dietary variable most strongly associated with Hb in this model. We emphasize that this is a statistical association in cross‐sectional data; it does not indicate that fat composition determines Hb levels nor that it outweighs unmeasured clinical drivers such as transfusion adequacy, iron overload, chelation, and disease severity, which are likely to be the dominant influences on Hb levels in thalassemia and were not captured in this study. The absence of association for BMI, junk‐food intake, and blood group also merits comment: the near‐universal junk‐food consumption (98.9%) left little variability against which an effect could be detected; BMI may be an insensitive marker of hematological status in a uniformly undernourished cohort; and blood group has no established mechanistic link to Hb concentration in thalassemia, consistent with the null finding observed here. More broadly, the associations reported here should be read against the confounders that this study could not address, such as disease severity, genotype, transfusion frequency and interval, iron overload status, chelation therapy, socioeconomic position, and total dietary intake, all of which plausibly influence Hb levels and may confound the dietary associations observed.

This study had several strengths. First, a relatively large sample of 270 participants drawn from clinical settings in Bangladesh strengthens our outcomes. However, as no a priori sample size calculation was performed, the statistical power was not reported. Second, the comprehensive and multidimensional approaches used to capture demographic, clinical, dietary, and lifestyle characteristics enabled a wider understanding of the factors associated with Hb levels in this population. Third, the linear regression model adjusted for potential confounders simultaneously, which improved the validity of the outcomes. However, despite these strengths, there are some limitations that need to be acknowledged. First, the cross‐sectional design allows only the evaluation of associations, not causal inference. Therefore, findings should be interpreted as associations, not as evidence that modifying trans‐fat or fruit intake would change Hb levels. Second, dietary intake information is self‐reported and is subject to recall bias and bias due to ‘flat slope syndrome’, which may result in under‐ or overestimation of dietary patterns. Third, dietary intake was captured through self‐report using categorical or binary indicators without quantitative recall or dietary biomarkers, which limits the robustness of the nutritional inferences. In particular, fruit intake was recorded only as a binary variable, and the very small proportion of nonconsumers may have resulted in unstable estimates. Fourth, participants were enrolled using convenience sampling from selected clinical settings, which may have introduced a selection bias and limited the generalizability of the results. Fifth, the cohort comprised predominantly children and adolescents, with adults under‐represented; therefore, the findings may not extend to adult patients with thalassemia. Finally, the study did not capture several clinical, biochemical, and genetic variables (e.g., thalassemia genotype, serum ferritin and iron status, chelation therapy, and transfusion history) known to influence Hb levels.

## 5. Conclusion

The current study highlights markedly low Hb levels, well below the recommended pretransfusion threshold, in individuals with thalassemia in Bangladesh. This is consistent with inadequate transfusion coverage and indicates a risk of progressive morbidity if not addressed. The comprehensive clinical and nutritional profiles of individuals with thalassemia revealed that the affected group was predominantly young, nutritionally vulnerable with a lower BMI, and carried a substantial burden of disease‐related complications. Hepatomegaly and splenomegaly were observed in almost all participants. The cohort exhibited a dietary pattern characterized by near‐universal junk food and trans‐fat intake, grossly inadequate vegetable consumption, and uniformly low levels of physical activity. The cross‐sectional design and poor overall dietary quality observed in the current study do not necessarily suggest a causal association between these factors. However, a clinical dietitian may play a responsible role in routine thalassemia care with structured nutritional counseling on trans‐fat and junk‐food intake and balanced fruit and vegetable intake. The findings should be used by policymakers and healthcare providers to enhance transfusion adequacy, according to international guidelines for pretransfusion Hb levels. There is a need for future longitudinal studies that include detailed dietary assessments and comprehensive biochemical data to better understand the causal pathways linking dietary behavior, nutritional status, and hematological outcomes in individuals with Thalassemia.

## Funding

This study did not receive any formal grants.

## Disclosure

All authors have read and approved the final version of this manuscript.

## Conflicts of Interest

The authors declare no conflicts of interest.

## Supporting Information

Additional supporting information can be found online in the Supporting Information section.

## Supporting information


**Supporting Information** Supporting Tables S1 and S2: Linear regression model assumptions and model sensitivity.

## Data Availability

Due to participant confidentiality and ethical restrictions, the data used in the current study are not readily available for sharing; however, upon reasonable request to the corresponding author, the data can be accessed after approval.
