# Supplementary material for: Clinical, Hematological, and Nutritional Profiling of Thalassemia Patients: Insights From a Cross‐Sectional Study
Source: Anemia. 2026 Sep 23;2026:8514650. doi: 10.1155/anem/8514650 (PMC13601856; doi:10.1155/anem/8514650)
Supplement: Supplementary file 1 [file ANEM-2026-8514650-s001.docx]

**Supplementary Table S1. Assumption diagnostics (full model)**

| **Assumption** | **Result** |
| --- | --- |
| Linearity / homoscedasticity | Breusch–Pagan LM = 13.28, p = 0.898 – satisfied |
| Normality of residuals | Shapiro–Wilk W = 0.948, p = 3.1 × 10⁻⁸ - violated (skew 1.08) |
| Multicollinearity | All VIF < 1.5 - satisfied |
| Influence | Participant 83 leverage = 1.000, Cook's D undefined |
| Missing data | None on any analysis variable |

*Because residual normality is violated, robust (HC3) standard errors and a 5,000-replicate bootstrap were used to check the fruit coefficient; both are reported in the Table 4 footnote.*

**Supplementary Table S2. Sensitivity analysis — model excluding the four near-invariant dietary variables (n = 270)**

| **Characteristic** | **β** | **95% CI** | **p-value** |
| --- | --- | --- | --- |
| Sex: Female (vs Male) | 0.14 | -0.19, 0.47 | 0.395 |
| Religion: Hindu (vs Islam) | 0.13 | -0.50, 0.76 | 0.690 |
| Blood group: A+ (vs B+) | -0.14 | -0.58, 0.30 | 0.523 |
| Blood group: AB− (vs B+) | -0.73 | -2.61, 1.15 | 0.446 |
| Blood group: AB+ (vs B+) | -0.49 | -1.13, 0.14 | 0.129 |
| Blood group: B− (vs B+) | -0.54 | -2.41, 1.34 | 0.575 |
| Blood group: O− (vs B+) | -1.11 | -3.02, 0.79 | 0.251 |
| Blood group: O+ (vs B+) | 0.16 | -0.24, 0.56 | 0.434 |
| Jaundice: Yes | -0.49 | -0.99, 0.02 | 0.059 |
| Hepatomegaly: Yes | 0.21 | -0.46, 0.89 | 0.535 |
| Splenomegaly: Yes | 0.06 | -0.44, 0.57 | 0.812 |
| Raw salt intake: Yes | 0.41 | -0.29, 1.11 | 0.255 |
| Daily fruit intake: Yes | **-0.76** | -1.31, -0.22 | **0.006** |
| Vegetable intake: Low (vs Moderate) | -0.51 | -1.19, 0.17 | 0.140 |
| Age, years | -0.01 | -0.02, 0.01 | 0.543 |
| BMI, kg/m² | 0.03 | -0.02, 0.08 | 0.246 |
| Meals per day | 0.26 | -0.83, 1.35 | 0.645 |

*Overall model: R² = 0.087; adjusted R² = 0.0254; F(17, 252) = 1.41; p = 0.131. Excluding trans fat, junk food, saturated fat and added sugar leaves the daily-fruit coefficient essentially unchanged (β = −0.76), indicating that the fruit association is not an artefact of the near-invariant predictors. Excluding participant 83 instead gives β = −0.77 (95% CI −1.31, −0.23; p = 0.006). However, the association is not significant unadjusted (Welch p = 0.064; Mann–Whitney p = 0.100), weakens under heteroscedasticity-robust HC3 standard errors (p = 0.037), and does not survive multiplicity correction.*
